# Gene–Dioxin Interactions and Pubertal Onset in Boys: Findings from the Russian Children’s Study

**DOI:** 10.1289/ehp.121-a30

**Published:** 2013-01-01

**Authors:** Julia R. Barrett

**Affiliations:** Julia R. Barrett, MS, ELS, a Madison, WI–based science writer and editor, has written for *EHP* since 1996. She is a member of the National Association of Science Writers and the Board of Editors in the Life Sciences.

Defining the genetic underpinnings of differences in human susceptibility to environmental exposures may help further refine the mechanism of action for a variety of agents. In a few cases, single-nucleotide polymorphisms (SNPs)—small differences in gene sequences between individuals—have been shown to underlie distinct biological responses. Investigators now report that dioxins may delay pubertal onset in boys, with stronger associations among boys with specific SNPs [*EHP* 121(1):111–117; Humblet et al.].

The investigators used data from the Russian Children’s Study, an ongoing prospective cohort study investigating gene–environment interactions affecting male pubertal development against a backdrop of environmental dioxin contamination. The cohort comprised mother–son pairs from Chapaevsk, Russia, a town severely contaminated by decades of industrial and agricultural chemical manufacturing. Dioxins are a class of long-lived pollutants linked to endocrine disruption and other adverse health effects. They are known to affect the transcription of numerous genes as well as cellular pathways controlling cell growth and death.

Boys had been recruited at age 8 or 9 years, and pubertal stage was assessed yearly based on testicular volume and genital maturity according to the Tanner Stages, a scale of physical development. Both the boys and their mothers provided blood samples for genetic and contaminant analyses at the study outset, and health, lifestyle, and diet data were collected through questionnaires.

**Figure:**
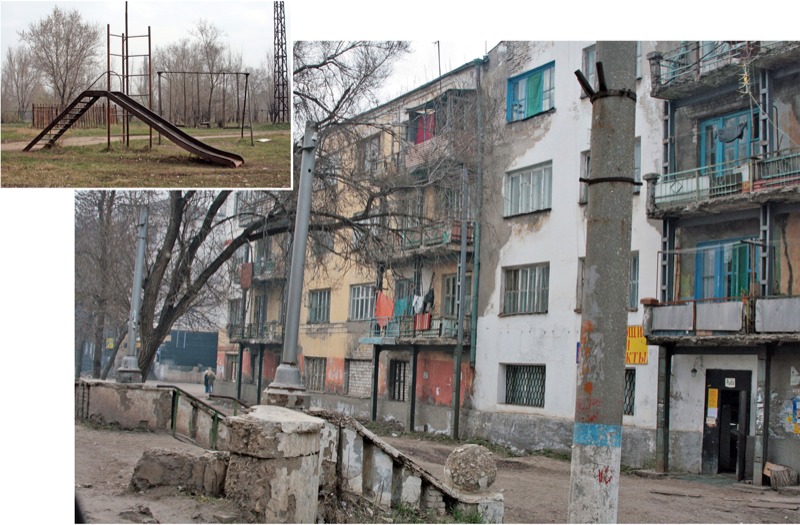
Several decades of industrial and agricultural chemical manufacturing left Chapaevsk severely contaminated with dioxins and dioxin-like chemicals. © Kommersant/zumapress.com

Previous analyses had indicated that serum concentrations of dioxins and dioxin-like compounds were associated with later pubertal onset among these boys. The current study, conducted when the boys had reached age 12 or 13 years, investigated whether genetic variations modified this outcome.

The investigation included 392 boys and involved 337 SNPs identified in 46 genes and 2 intergenic regions (stretches of DNA between protein-coding genes) that may influence pubertal onset or the biological response to dioxins. The association of serum dioxins with later pubertal onset was confirmed, and two statistical methods were used to examine gene-related modification of this response. The primary method identified 3 SNPs that significantly modified the association between dioxin exposure and timing of puberty; however, the association held only for genital maturity, not testicular volume, and it was not observed with the secondary statistical method.

The study has several limitations. First, findings may apply only to boys with high serum dioxin levels. Additional limitations arise from the low frequency of some SNPs in the population, the potential omission of other genes relevant to pubertal onset, and the assumption that the selected SNPs served as appropriate markers of susceptibility. Therefore, the implications of the observed gene–environment interactions are uncertain, and additional study is needed for clarification.

